# Comparative evaluation of the effects of four types of iron supplements on primary teeth discoloration: an in vitro study

**DOI:** 10.1186/s12903-025-06846-x

**Published:** 2025-09-24

**Authors:** Parisa Vahedi, Nikta Bagheri, Somayeh Khoramian Tusi, Manijeh Mohammadian

**Affiliations:** 1 Department of Pediatric Dentistry, Alborz University of Medical Sciences, Karaj, Iran; 2 Student Research Committee, Alborz University of Medical Sciences, Karaj, Iran; 3 Department of Dental Biomaterials, Iran University of Medical Sciences, Tehran, Iran

**Keywords:** Iron compounds, Tooth discoloration, Primary teeth

## Abstract

**Background:**

Iron supplements can cause discoloration in children’s teeth, which can impact their confidence and social interactions. This staining may discourage parents from continuing treatment. The study compared different types of iron supplements—encapsulated (liposomal, sucrosomial) and non-encapsulated (ferrous sulfate, iron polysaccharide complex)—to assess their impact on tooth discoloration.

**Materials and methods:**

In this in vitro study, a total of 65 intact anterior primary teeth were examined. Their initial color was assessed using an X-RITE spectrophotometer in the CIELab system. The control group was treated with normal saline, while experimental groups were exposed to iron supplements: Liposofer, Amivital Ferrous Sulphate, SiderAl, and Feramax. The teeth were immersed in these solutions for two weeks before final color measurements. SPSS software version 27 was used for statistical analysis, including paired t-tests, one-way ANOVA, LSD multiple comparison tests, Tamhane’s T2 test, and the Shapiro-Wilk test. The level of statistical significance was set at *P* < 0.05.

**Results:**

The mean ∆E parameter (color assessment) of primary teeth was significantly higher in the groups treated with Amivital Ferrous Sulphate and SiderAl iron supplements compared to the other groups (*P* < 0.05). Among the iron supplements, Liposofer and Feramax showed the lowest mean ∆E parameter for primary teeth (*P* < 0.05).

**Conclusion:**

The type of iron supplement may play a key role in primary tooth discoloration. These findings suggest that polysaccharide and liposomal iron supplements may cause less discoloration in vitro, though further clinical research is needed to confirm these findings.

## Background

Iron deficiency is one of the most common nutritional deficiencies, affecting over two billion people. Iron plays vital roles in the body, such as supporting myoglobin activity, facilitating cellular respiration, and aiding energy production [1]. 

Iron deficiency anemia (IDA) is a significant concern, particularly for children and women of reproductive age, especially in lower-income countries. Young children, especially those under five, are more vulnerable due to rapid growth demands [2–4]. Iron supplementation, either orally, intravenously, or through food fortification, is a common approach to managing iron deficiency. For children under five, iron salts are frequently administered in the form of drops or syrups, often fortified with folic acid and/or vitamin B_12_ [5]. However, oral iron supplements are recommended as the first line of treatment before considering intravenous methods due to lower risks, despite the oral route being slightly less effective [6, 7]. 

According to global public health recommendations, in areas with a high prevalence of anemia, daily iron supplementation is recommended for infants aged 6–23 months, typically at a dose of 10-12.5 mg of elemental iron per day to prevent iron deficiency anemia [8]. However, inadequate or irregular iron supplementation is frequently reported [9]. 

The most commonly prescribed iron supplement is ferrous sulfate, as it is simple and cost-effective. Other iron forms, such as polysaccharide iron complexes and newer formulations like liposomal or sucrosomial iron supplements, have been developed to address treatment challenges and side effects [10]. 

Common side effects of iron supplements include gastrointestinal discomfort, nausea, extrinsic tooth discoloration, adverse effects on enamel microhardness, and an unpleasant metallic taste [11–14]. Tooth discoloration, in particular, often concerns parents as it may impact children’s confidence and social interactions [13]. 

Technologies like encapsulation, including liposomal and sucrosomial iron, aim to reduce these side effects [15]. Liposomal iron improves absorption in the intestines while minimizing direct contact with the teeth and gastrointestinal lining [16]. Similarly, sucrosomial iron enhances bioavailability and gastrointestinal tolerance by encapsulating ferric pyrophosphate in a phospholipid matrix [17]. 

Iron polysaccharide complexes are an alternative to traditional iron salts. They consist of iron ions bound to a polysaccharide carrier, with Fe^3+^ as the aglycone. Since Fe^3+^ does not exist freely, these supplements minimize intestinal discomfort. Gastric fluid breaks them down, releasing iron and saccharides, which help convert Fe^3+^ to Fe^2+^. Once reduced to Fe^2+^, the iron is absorbed and utilized by the body [10, 18]. 

Conducting the study in vitro allowed for a precise assessment of the discoloration potential of iron supplements. Various methods have been employed in previous studies to quantify tooth discoloration, ranging from subjective visual assessments using shade guides to objective colorimetric techniques. Among these, spectrophotometry based on the CIELAB color system is considered a reliable and reproducible approach. This system evaluates color through three parameters (L, a, b) and computes the overall color change as ΔE. Studies such as Babaei et al. [19] and Pani et al. [20] have also adopted this method, highlighting its accuracy in detecting minor color changes. Given its objectivity and sensitivity, this method was selected for the present study to assess the staining potential of iron supplements on primary teeth.

Apart from the deposition of ferric ions, tooth discoloration may also be influenced by surface roughness, protein-mineral interactions, and other chemical properties of the formulations. It is claimed that liposomal, sucrosomial, and polysaccharide iron complexes may reduce common side effects, such as tooth discoloration, more effectively than standard ferrous sulfate. This study aimed to compare these four types of iron supplements to assess their effects on primary tooth discoloration. The null hypothesis of the study was that there would be no significant difference in tooth discoloration among the four iron supplements.

## Materials and methods

This in vitro study was approved by the Ethics Committee of Alborz University of Medical Sciences. (îApproval code: IR.ABZUMS.REC.1403.230)

This experimental study investigated the effects of four iron supplements on the discoloration of primary teeth (Table 1).

**Table 1:** Understudy iron supplements

Iron Supplement	Type Of Iron Supplement	Ingredients	Company/Country
Liposofer	Liposomal	30 ml of iron drops, with each ml containing 7 mg of iron.	BSK/Iran
Amivital Ferrous Sulphate	Ferrous Sulphate	15 mL of iron drops, with each mL containing 125 mg of ferrous sulfate heptahydrate, equivalent to 25 mg of iron.	Amin Pharma/Iran
SiderAl	Sucrosomial	30 mL iron drops with a 9.1 g sachet, where each mL contains 7 mg iron	Junia Pharma/Italy
Feramax	Polysaccharide iron complex	41.5 g iron powder (60 scoops), each scoop containing 15 mg iron	BioSyent Pharma/Canada

The sample size was calculated to be 13 teeth in each group (a total of 65 teeth for the control and experimental groups) according to a previous study by Babaei et al. [19], assuming α = 0.05 and β = 0.10.

The samples included primary teeth that were either naturally exfoliated or extracted for orthodontic purposes. After screening based on the study criteria (no signs of decay, fractures, cracks, restorations, or enamel hypoplasia), 65 teeth were randomly chosen. They were initially stored in normal saline at 37 °C, then disinfected in 0.5% chloramine T for one week and polished using a low-speed handpiece, brush, and prophylaxis paste. The teeth were stored in normal saline until examination.

On the day of analysis, the teeth were removed from the normal saline and dried.

A box with individual compartments was used to house the samples, with each compartment containing one sample. The teeth were randomly assigned to a compartment for baseline color measurement using a manual draw method. Each compartment was labeled for identification.

The control group was immersed in normal saline. Although a positive control (such as a known discoloring agent) was not included, the use of normal saline as a negative control allowed for direct assessment of each iron supplement’s relative effect on tooth color under identical conditions. This design enabled us to isolate the discoloration potential of the formulations themselves, without background interference. The experimental groups were exposed to iron supplement solutions. Each solution contained 100 mg of pure iron in 250 mL of normal saline, prepared as follows: 14.3 mL Liposofer, 4 mL Amivital Ferrous Sulphate, 14.3 mL SiderAl, 4.6 g Feramax.

The SiderAl supplement was prepared by dissolving the sachet powder in its solution, after which 14.3 mL of the mixture was extracted and added to 250 mL of normal saline.

Each tooth was stabilized on a standard gray plate using wax, and its baseline color was assessed on the buccal surface using a Ci6X spectrophotometer (X-RITE, USA). Blinding was not applied during color measurements; however, as color changes were assessed using an objective digital spectrophotometer, the potential impact of observer bias was minimized. All measurements were conducted under identical lighting conditions at the same location to ensure consistency. Baseline color values (L, a, b) were recorded prior to immersion and used to confirm similarity among groups. The teeth were then returned to their slots and submerged in 5 mL of the prepared solutions.

After two weeks, the samples were removed, rinsed with normal saline, and dried. The 14-day immersion period was chosen to reflect cumulative exposure for comparative analysis, not direct clinical simulation. Color changes were analyzed using the CIELab system, which defines color in a three**-**dimensional space based on the following components:

$${\text{L}}\,{\text{parameter}} \to {\text{Lightness }}(0\,=\,{\text{black}},\,{\text{1}}00\,=\,{\text{white}})$$

$$\begin{gathered} {\text{a}}\,{\text{parameter}} \to {\text{Red}} - {\text{green}} \hfill \\ \,{\text{axis}}({\text{positive}}\,=\,{\text{red}},\,{\text{negative}}\,=\,{\text{green}}) \hfill \\ \end{gathered} $$

$$\begin{gathered} {\text{b}}\,{\text{parameter}} \to {\text{Yellow}} - {\text{blue}} \hfill \\ \,{\text{axis}}({\text{positive}}\,=\,{\text{yellow}},\,{\text{negative}}\,=\,{\text{blue}}) \hfill \\ \end{gathered} $$

Spectrophotometer readings for L, a, and b were recorded for each sample.

The degree of discoloration (ΔE) was determined using the following equation:

$$\Delta E\,{\left[ {{{\left( {\Delta a} \right)}^2}\,+\,{{\left( {\Delta b} \right)}^2}\,+\,{{\left( {\Delta L} \right)}^2}} \right]^{\frac{1}{2}}}$$

All data were analyzed using SPSS version 27. Comparisons between baseline and post-exposure color parameters were performed using a paired t-test. Differences among experimental groups were assessed using one-way ANOVA, followed by multiple comparison tests: LSD test (for equal variances), Tamhane’s T2 test (for unequal variances). Cohen’s d or η² were also calculated and reported as measures of effect size.

The Shapiro-Wilk test was used to assess the data distribution. Levene’s test was conducted to ensure the homogeneity of variances. Statistical significance was set at *p* < 0.05.

## Results

The skewness and kurtosis values of the tooth color parameters in all study groups, both at baseline and after two weeks, were within the range of − 2 to + 2, indicating a normal distribution of the assessed variables. Furthermore, the results of the Shapiro–Wilk test showed that the p-values for all tooth color parameters in the study groups, at both time points, were greater than 0.05. Therefore, the assumption of normal distribution was met.

As shown in Table 2, the paired t-test indicated that the mean of the L and a parameters of the primary teeth significantly decreased two weeks after immersion in the solution containing normal saline compared to the beginning of the study (*P* < 0.001 and *P* = 0.001, respectively), while the mean of the b parameter of the primary teeth did not show a significant decrease (*P* = 0.111).

**Table 2:** Baseline vs. post-immersion color of teeth in normal saline group

Parameter	*N*	Minimum	Maximum	Skewness	Kurtosis	Mean	Standard Deviation	95% CI of Difference	Cohen’s d	*P*-value
L At baseline	13	73.99	85.99	0.183	0.613	79.67	3.13	2.05, 2.92	0.72	< 0.001
L After two weeks	13	72.19	82.98	0.258	0.231	77.18	2.87			
a At baseline	13	− 0.43	1.23	− 0.299	0.439	0.52	0.45	0.18, 0.49	0.26	0.001
a After two weeks	13	− 0.88	0.87	− 0.566	− 0.184	0.18	0.52			
b At baseline	13	9.57	16.16	− 0.760	1.541	13.51	1.68	− 0.16, 1.36	1.26	0.111
b After two weeks	13	11.09	14.95	0.193	− 1.297	12.91	1.43			

As shown in Table 3, the paired t-test indicated that the mean of the L and b parameters of the primary teeth significantly decreased two weeks after immersion in the solution containing the Liposofer iron supplement compared to the beginning of the study (*P* = 0.001 and *P* = 0.004, respectively), while the mean of the a parameter of the primary teeth did not show a significant decrease (*P* = 0.148).

**Table 3:** Baseline vs. Post-Immersion color of teeth in liposofer group

Parameter	*N*	Minimum	Maximum	Skewness	Kurtosis	Mean	Standard Deviation	95% CI of Difference	Cohen’s d	*P*-value
L At baseline	13	70.60	86.72	− 1.550	1.427	81.95	4.28	3.29, 9.64	5.25	0.001
L After two weeks	13	63.84	80.87	− 1.175	1.104	75.48	5.01			
a At baseline	13	− 0.39	1.76	0.436	− 0.532	0.55	0.67	− 0.14, 0.83	0.81	0.148
a After two weeks	13	− 0.73	2.57	1.440	1.328	0.20	0.93			
b At baseline	13	11.51	22.94	1.132	0.343	15.56	3.77	0.51, 2.13	1.34	0.004
b After two weeks	13	9.29	22.58	0.782	0.342	14.24	3.86			

As shown in Table 4, the paired t-test indicated that the mean of the L parameter of the primary teeth significantly decreased two weeks after immersion in the solution containing the Amivital Ferrous Sulphate iron supplement compared to the beginning of the study (*P* < 0.001), while the mean of the a and b parameters of the primary teeth showed a significant increase (*P* = 0.002 and *P* < 0.001, respectively).

**Table 4:** Baseline vs. Post-Immersion color of teeth in amivital ferrous sulphate group

Parameter	*N*	Minimum	Maximum	Skewness	Kurtosis	Mean	Standard Deviation	95% CI of Difference	Cohen’s d	*P*-value
L At baseline	13	65.41	84.87	− 1.355	1.492	78.65	5.50	5.52, 12.07	5.42	< 0.001
L After two weeks	13	55.23	76.43	− 1.429	1.212	69.86	5.58			
a At baseline	13	− 0.35	3.39	0.837	0.827	0.89	1.08	− 1.578, − 0.45	0.93	0.002
a After two weeks	13	− 0.48	4.83	0.176	0.122	1.91	1.47			
b At baseline	13	8.50	19.35	0.422	0.068	13.03	3.12	− 6.31, − 2.86	2.89	< 0.001
b After two weeks	13	10.33	24.13	-0.095	− 0.451	17.62	3.82			

As shown in Table 5, the paired t-test indicated that the mean of the L and b parameters of the primary teeth significantly decreased two weeks after immersion in the solution containing the SiderAl iron supplement compared to the beginning of the study (*P* < 0.001 and *P* = 0.008, respectively), while the mean of the a parameter of the primary teeth did not show a significant increase (*P* = 0.716).

**Table 5:** Baseline vs. Post-Immersion color of teeth in sideral group

Parameter	*N*	Minimum	Maximum	Skewness	Kurtosis	Mean	Standard Deviation	95% CI of Difference	Cohen’s d	*P*-value
L At baseline	13	75.50	87.92	− 0.817	− 0.301	82.90	3.94	6.35, 11.74	4.46	< 0.001
L After two weeks	13	66.52	82.71	0.048	− 0.739	73.86	4.73			
a At baseline	13	− 0.42	1.82	1.030	1.076	0.37	0.64	− 0.14, 0.85	0.82	0.716
a After two weeks	13	− 0.77	2.62	1.444	1.152	0.70	1.02			
b At baseline	13	8.64	16.51	− 0.382	− 0.217	13.17	2.20	0.49, 2.67	1.80	0.008
b After two weeks	13	8.74	13.78	− 0.401	− 0.789	11.59	1.71			

As shown in Table 6, the paired t-test indicated that the mean of the L parameter of the primary teeth significantly decreased two weeks after immersion in the solution containing the Feramax iron supplement compared to the beginning of the study (*P* = 0.003), while the mean of the a parameter of the primary teeth significantly increased (*P* = 0.024). However, the mean of the b parameter of the primary teeth did not show a significant decrease two weeks after immersion in the solution containing the Feramax iron supplement compared to the beginning of the study (*P* = 0.232).

**Table 6:** Baseline vs. Post-Immersion color of teeth in Feramax group

Parameter	*N*	Minimum	Maximum	Skewness	Kurtosis	Mean	Standard Deviation	95% CI of Difference	Cohen’s d	*P*-value
L At baseline	13	63.27	87.64	− 1.010	− 0.122	79.43	7.82	2.04, 7.82	4.78	0.003
L After two weeks	13	62.54	83.85	− 0.441	− 0.309	74.50	6.10			
a At baseline	13	− 0.55	1.46	0.687	− 0.180	0.26	0.66	− 0.55, − 0.05	0.42	0.024
a After two weeks	13	− 0.40	1.58	0.014	− 0.999	0.56	0.62			
b At baseline	13	9.37	17.23	− 0.287	− 0.974	13.49	2.56	− 0.43, 1.61	1.69	0.232
b After two weeks	13	10.04	14.91	− 0.619	− 0.243	12.90	1.38			

Levene’s statistical test showed that the variance of the L parameter (test statistic = 1.015, df = 4 and 60, *P* = 0.296), the variance of the a parameter (test statistic = 2.121, df = 4 and 60, *P* = 0.090), and the variance of the b parameter (test statistic = 2.156, df = 4 and 60, *P* = 0.085) of the primary teeth at the beginning of the study had no statistically significant difference among the study groups. In other words, the variance of the color parameters of the primary teeth at the beginning of the study is homogeneous among the study groups, and therefore the assumption of homogeneity of variances is met.

The one-way analysis of variance showed that the mean of the color parameters of the primary teeth at the beginning of the study did not have a statistically significant difference among the study groups (*P* > 0.05). In other words, the mean of the color parameters of the primary teeth at the beginning of the study is homogeneous among the study groups.

Levene’s statistical test showed that the variance of the L parameter (test statistic = 1.607, df = 4 and 60, *P* = 0.184), the variance of the a parameter (test statistic = 1.802, df = 4 and 60, *P* = 0.148), and the variance of the b parameter (test statistic = 1.726, df = 4 and 60, *P* = 0.163) of the primary teeth two weeks after immersion in the solution had no statistically significant difference among the study groups. In other words, the variance of the color parameters of the primary teeth two weeks after immersion in the solution is homogeneous among the study groups, and therefore the assumption of homogeneity of variances is met.

The one-way analysis of variance showed that the mean of the color parameters of the primary teeth two weeks after immersion in the solution has a statistically significant difference among the study groups (*P* < 0.05). The Least Significant Difference (LSD) multiple comparisons test showed that two weeks after immersion in the solution:

- The mean of the L parameter of the primary teeth, due to the Amivital Ferrous Sulphate iron supplement, is significantly lower than in the other groups (*P* < 0.05), while the other groups did not show a statistically significant difference with each other (*P* > 0.05).
- The mean of the a parameter of the primary teeth, due to the Amivital Ferrous Sulphate iron supplement, is significantly higher than in the other groups (*P* < 0.001), while the other groups did not show a statistically significant difference with each other (*P* > 0.05).
- The mean of the b parameter of the primary teeth, due to the Amivital Ferrous Sulphate iron supplement, is significantly higher than in the other groups (*P* < 0.001), while the other groups did not show a statistically significant difference with each other (*P* > 0.05).

As shown in Table 7, the skewness and kurtosis values of the ΔE parameter of the primary teeth two weeks after immersion in the solution are within the range of -2 to + 2, which indicates a normal distribution for the ΔE parameter. Also, the results of the Shapiro-Wilk statistical test showed that the P-value for the ΔE parameter of the primary teeth two weeks after immersion in the solution is greater than 0.05 for each of the study groups, and therefore the ΔE parameter has a normal distribution in each of the study groups (*P* > 0.05). Thus, the assumption of normal distribution of the data is met.

**Table 7:** Descriptive characteristics of ΔE after two weeks of immersion in the solution by study groups

Parameter	*N*	Minimum	Maximum	Mean	Standard Deviation	Skewness	Kurtosis	95% CI	η^2^	*P*-value
Normal Saline	13	1.56	4.35	2.85	0.75	0.043	0.372	2.40, 0.30	0.43	< 0.001
Liposofer	13	2.01	18.97	7.11	4.74	1.381	1.197	4.25, 9.98		
Amivital Ferrous Sulphate	13	7.62	18.80	11.22	3.10	1.258	1.786	9.35, 13.10		
SiderAl	13	4.31	18.05	9.52	4.10	0.847	0.264	7.04, 12.00		
Feramax	13	2.94	14.18	6.28	3.17	1.476	1.148	4.36, 8.19		

To determine the magnitude of the observed changes within each group, Cohen’s d was calculated as a measure of effect size. The analysis revealed a very large effect size for the change in lightness (L) in the Amivital Ferrous Sulphate group (d = 5.42) and the SiderAl group (d = 4.46), indicating a substantial and practically significant darkening of the teeth. The Liposofer and Feramax groups also showed large effect sizes (d = 5.25 and d = 4.78, respectively), though the most pronounced changes in the red-green (a) and yellow-blue (b) axes were observed in the Amivital Ferrous Sulphate group. In contrast, the control group exhibited a much smaller effect size for all color changes, confirming that the discoloration in the experimental groups was primarily due to the supplements themselves.

Levene’s statistical test showed that the variance of the ΔE parameter of the primary teeth (test statistic = 3.347, df = 4 and 60, *P* = 0.015), two weeks after immersion in the solution, has a statistically significant difference among the study groups. In other words, the variance of the ΔE parameter of the primary teeth two weeks after immersion in the solution is not homogeneous among the study groups, and therefore the assumption of homogeneity of variances is not met.

As shown in Table 2, one-way ANOVA analysis revealed that the mean ΔE parameter of primary teeth two weeks after immersion in the solution exhibited a statistically significant difference among the examined groups (*P* < 0.001). Specifically, the multiple com parisons test using Tamhane’s T2 indicated that two weeks after immersion in the solution: The mean ΔE parameter of primary teeth exposed to Amivital Ferrous Sulphate and SiderAl iron supplements was significantly higher than in other groups (*P* < 0.05). The mean ΔE parameter of primary teeth exposed to normal saline was significantly lower than in other groups (*P* < 0.05). The mean ΔE parameter of primary teeth exposed to Liposofer and Feramax iron supplements fell within an intermediate range (P< 0.05).

One-way ANOVA analysis revealed that the mean ΔE parameter of primary teeth two weeks after immersion in the solution exhibited a statistically significant difference among the examined groups (*P* < 0.001). Specifically, the multiple comparisons test using Tamhane’s T2 indicated that two weeks after immersion in the solution. The mean ΔE parameter of primary teeth exposed to Amivital Ferrous Sulphate and SiderAl iron supplements was significantly higher than in other groups (*P* < 0.05). The mean ΔE parameter of primary teeth exposed to normal saline was significantly lower than in other groups (*P* < 0.05). The mean ΔE parameter of primary teeth exposed to Liposofer and Feramax iron supplements fell within an intermediate range (*P* < 0.05).

To further quantify the impact of the supplement type on overall discoloration, the effect size was calculated using eta-squared (η2). The result (η2 = 0.43) indicates a large effect size. This means that 43% of the total variance in tooth discoloration (ΔE) can be attributed to the specific type of iron supplement used. This powerful finding underscores that the choice of supplement is a major factor in the extent of tooth staining.

## Discussion

The present in vitro study aimed to evaluate the extent of color change in primary teeth following exposure to four types of iron supplements: ferrous sulfate, liposomal, sucrosomial, and polysaccharide iron complexes. These four types of iron supplements are the most commonly consumed iron supplements in Iran. The issue is clinically significant, particularly in pediatric dentistry, where esthetic changes in teeth may influence treatment acceptance among parents and cooperation from children.

Since visual assessment of discoloration can be influenced by lighting conditions, material properties, and psychological factors, a spectrophotometer was employed in this study as one of the most accurate devices for dental color measurement [21]. This instrument quantifies color based on the CIELab system.

The ΔE parameter reflects the magnitude of color change over time. Values above 1 are considered perceptible, and changes up to 3.7 are generally acceptable [22, 23]. In the current study, the mean ΔE in all experimental groups—except normal saline—exceeded 3.7, indicating a clinically noticeable level of discoloration caused by iron supplements. Nevertheless, a receiver-operating-characteristic (ROC) analysis was not conducted to derive a study-specific ΔE threshold, and this is only based on previous studies.

The L (lightness) value decreased across all groups, especially in the Amivital Ferrous Sulphate and SiderAl groups, suggesting a general darkening of tooth surfaces. The a parameter (red-green axis) increased significantly in the Ferrous Sulphate, SiderAl, and Feramax groups (a shift toward red), while it decreased in the normal saline and Liposofer groups. Regarding the b axis (yellow-blue), a significant reduction (shift toward blue) was observed in the SiderAl and Liposofer groups, whereas a notable increase (toward yellow) occurred in the Ferrous Sulphate group. These findings revealed that Amivital Ferrous Sulphate induced the greatest degree of discoloration, followed by SiderAl. In contrast, Feramax and Liposofer were associated with much lower levels of color change. This highlights the role of supplement formulation and iron delivery systems in influencing enamel discoloration.

The present findings are supported by Babaei et al. [19], who examined the relationship between pH and viscosity of iron drops and their staining effect on primary teeth. They reported that ferrous sulfate-based supplements caused more significant discoloration, while liposomal formulations such as Liposofer resulted in less visible staining likely due to its encapsulated structure, which reduces direct contact between iron ions and enamel surfaces. Similarly, Nazemisalman et al. [24] investigated the effect of iron salts on primary enamel under both healthy and cariogenic conditions. Their study showed that discoloration was more pronounced in demineralized enamel, highlighting the increased susceptibility of weakened tooth surfaces to staining. They also noted that such discoloration could be mistaken for caries by parents, potentially leading to treatment refusal or poor adherence. Together, these studies emphasize the influence of both supplement properties and enamel condition on the severity of discoloration observed in clinical and subclinical scenarios. Since primary tooth enamel has less mineralization than permanent tooth enamel, has more pore volume, and its calcified layer is thinner and more irregular, it is more prone to adverse effects such as dental wear and surface staining due to frequent use of low-pH supplements.

Findings from the present study are also aligned with those of Abbasi et al. [25], who showed that nano-encapsulated iron supplements caused significantly less discoloration compared to conventional ferrous sulfate drops such as Irofant and Feroglobin. Both studies emphasized the protective effect of encapsulation technologies in limiting surface staining, particularly in sound enamel.

Similarly, Pani et al. [20] reported enamel discoloration following exposure to iron-based compounds, particularly ferric salts. Although the formulations in their study differed from those examined here, the general pattern of iron-induced staining is consistent with the present findings.

In contrast, the results differ from those reported by Tayebi et al. [26], who did not observe significant differences in tooth discoloration among various iron supplements. This discrepancy could be attributed to their use of chemically similar ferrous sulfate formulations across all groups and the shorter evaluation period (up to 96 h), which may have been insufficient to detect cumulative effects. Ferrous sulfate is one of the most commonly used iron supplements, widely consumed due to its low cost and availability. However, one of its known side effects is tooth discoloration. The primary cause of this discoloration is the chemical reaction between the iron in ferrous sulfate and the minerals present in teeth. Ferrous sulfate can cause tooth discoloration, which typically turns black or dark brown. The exact mechanism of this discoloration is not fully understood, but it is believed that this process involves the formation of iron compounds, such as ferric sulfide, when iron reacts with sulfur compounds in the mouth [27]. This reaction can lead to the deposition of these compounds on the tooth surface, resulting in discoloration. The iron in these supplements can react with tooth enamel and create dark stains on teeth. These stains are difficult to remove and may require dental procedures for elimination. While detailed elucidation of the encapsulation or binding mechanisms of liposomal, sucrosomial, and polysaccharide formulations is beyond the scope of this in vitro study, these technologies are designed to reduce the release of free iron ions and, consequently, limit direct iron–enamel contact and staining.

A key strength of this study was the standardization of iron concentrations across all groups (100 mg in 250 mL), which enabled fair comparisons. Furthermore, the use of spectrophotometric analysis in the CIELab color space provided objective and precise color measurements. The ΔE value served as a reliable index for quantifying clinically relevant discoloration and facilitated accurate intergroup comparisons. While most previous studies have focused solely on ΔE as a measure of tooth discoloration, the present study also analyzed the individual L, a, and b components to determine the direction of color changes. This additional analysis revealed alterations in brightness and hue, offering a more detailed understanding of the visual impact of iron supplements on primary teeth. These findings may assist in clarifying the esthetic implications beyond total color shift. Also, the inclusion of effect size calculations (Cohen’s d and η2), which move beyond mere statistical significance (p-value) to assess the practical significance of the findings. While the p-value confirms that the differences between groups are unlikely to be due to chance, the large effect sizes reported here demonstrate that these differences are also substantial in magnitude. For example, the eta-squared value revealed that 43% of the discoloration was directly attributable to the type of iron supplement, a finding of considerable clinical importance. This provides robust evidence that the formulation of an iron supplement is a critical determinant of its staining potential and reinforces the recommendation to consider encapsulated forms like liposomal or polysaccharide iron to minimize this undesirable side effect.

Nonetheless, certain limitations should be acknowledged. As an in vitro study, this experiment could not replicate the dynamic oral environment, including salivary flow, biofilm formation, and pH fluctuations, which are known to affect tooth discoloration in vivo. Additionally, physicochemical characteristics of the iron formulations, such as pH, viscosity, and titratable acidity, were not measured, although they may influence staining potential. Furthermore, the staining process may be affected not only by the type and concentration of iron, but also by interactions with enamel proteins, the ionic properties of the formulation, and surface porosity. These factors were not isolated in the current study and merit investigation in future work. It should be noted that a formal correction for multiple comparisons (e.g., Bonferroni or Tukey’s HSD) was not applied to the post-hoc tests in this study. While the very large effect sizes and highly significant p-values suggest that the primary findings are robust, the lack of multiplicity control is a statistical limitation. Therefore, the results of the pairwise comparisons should be interpreted with a degree of caution, and future studies are encouraged to replicate these findings using more stringent statistical approaches. Further in vivo research is recommended to validate these observations under clinical conditions.

## Conclusion

Within the limits of this in vitro study, the type of iron supplement significantly influenced primary tooth discoloration. The Amivital Ferrous Sulphate and SiderAl groups exhibited the highest color change values, while Liposofer and Feramax showed lower discoloration levels. However, pairwise comparisons indicated no statistically significant difference between Ferrous Sulphate and SiderAl, or between Feramax and Liposofer.

## Data Availability

The datasets analyzed during the current study are available from the corresponding author on reasonable request.
